# Rotating Shift Work and Burnout Among Nurses in Hebron Hospitals, West Bank, Palestine: A Cross‐Sectional Study

**DOI:** 10.1002/puh2.70172

**Published:** 2025-12-26

**Authors:** Yousef Jaradat, Mohammed Qtait, Nesreen Alqaissi, Dana Abo Omer, Asal Atawneh, Areen Abu Sharkh, Mohammed Baradia, Hamza A. Drabee

**Affiliations:** ^1^ Nursing College Palestine Polytechnic University Hebron Palestine

**Keywords:** burnout, healthcare workers, Maslach Burnout Inventory, Palestinian nurses, rotating shifts, shift work

## Abstract

This study examines the relationship between rotating shift work and burnout among nurses in Hebron hospitals within the context of Palestine's unique political and social conditions. A cross‐sectional survey was conducted from January to March 2025 among registered nurses using a structured questionnaire that captured socio‐demographic and workplace characteristics. Burnout was assessed using the Maslach Burnout Inventory (MBI). The findings revealed that nurses working rotating shifts reported significantly higher levels of burnout, with female nurses experiencing greater emotional exhaustion and reduced personal accomplishment (PA). Unmarried nurses and those without children demonstrated elevated depersonalization (DP), low PA, and overall burnout. The highest burnout levels were observed at Hebron Alia Governmental Hospital. Additionally, nurses with less than 7 years of experience exhibited significantly higher DP and lower PA. These results underscore the urgent need for targeted interventions, including optimizing staffing, providing mental health support, and fostering a supportive work environment. Given the cross‐sectional nature and reliance on self‐reported data, the findings should be interpreted with caution. Further research is recommended to explore the long‐term effects of shift work on patient care quality.

## Graphical abstract

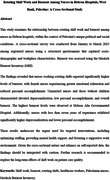

## 1 Introduction

Nursing is a profession often associated with shift work, long hours, and high emotional demands, all of which contribute to elevated risks of occupational stress and burnout [1]. Burnout is a work‐related syndrome resulting from prolonged exposure to physical, psychological, and emotional stressors in the workplace [2]. It is typically characterized by three core dimensions: emotional exhaustion (EE), depersonalization (DP), and reduced personal accomplishment (PA).

Globally, burnout among nurses is a serious concern. Studies estimate that between 25% and 37% of nurses in the United States and Europe experience significant levels of burnout; burnout has far‐reaching consequences, including increased absenteeism, higher staff turnover, and compromised patient safety—such as medication errors and adverse events [1].

Numerous studies have explored the impact of shift characteristics—especially 12‐h shifts—on nurse burnout. Findings from large cross‐sectional studies suggest that longer shifts are associated with higher EE compared to traditional 8‐h shifts [3, 4]. However, other research indicates that when nurses voluntarily choose 12‐h shifts, the negative effects may be mitigated due to increased autonomy, a concept supported by the job demands–resources (JD–R) model [5, 6].

The JD–R model explains burnout as a consequence of excessive JDs (e.g., long hours, emotional stress) and insufficient job resources (e.g., autonomy, support). Autonomy, in particular, has been linked to better job satisfaction, improved sleep quality, and reduced fatigue [7]. Nonetheless, these protective factors have not been adequately explored in the nursing field.

Night shift work, especially rotating schedules, poses additional challenges by disrupting circadian rhythms, reducing recovery time, and affecting work–life balance. Rotational shifts are thought to impose greater psychological and physiological strain than fixed shifts [8]. Yet, few studies have specifically examined the impact of rotation, as opposed to shift length alone, on nurse burnout.

In the Palestinian healthcare context, the evidence remains limited and sometimes contradictory. For instance, Hamdan and Hamra [9] found higher burnout levels in West Bank emergency departments than in Gaza, possibly due to regional differences in healthcare delivery. Conversely, Abukhader et al. [10] found higher EE among nurses working fixed day shifts, contradicting broader findings and emphasizing the need for contextual research.

Demographic factors also play a role. Younger, unmarried nurses and those without children often report higher burnout levels, potentially due to limited coping strategies or professional experience [10, 11]. In contrast, female nurses may be more prone to EE due to dual professional and familial roles [12, 13]. However, other studies report that male nurses experience more DP [14], indicating a gendered experience of burnout.

Despite growing awareness, significant gaps remain in understanding the nuanced impact of shift type, workplace environment, and demographic factors—especially in politically unstable and resource‐limited settings like Palestine. Most studies to date rely on cross‐sectional designs and self‐reported data, limiting causal interpretation and generalizability [15].

Therefore, the current study aims to investigate the association between rotating shift work and burnout among nurses in Hebron hospitals, considering socio‐demographic and workplace predictors.

## 2 Literature Review

Burnout, first conceptualized by Freudenberger [16], is now recognized as a psychological syndrome that results from chronic occupational stress, particularly in human service professions like nursing [2]. It is typically defined by three dimensions: EE, DP, and reduced PA. Nurses are particularly vulnerable due to the emotionally intense nature of their work and frequent exposure to stressful clinical environments [17].

A growing body of literature has explored the prevalence and predictors of burnout among nurses. In the Middle East, a systematic review by Chemali et al. [18] involving over 10,000 nurses reported consistently high burnout rates, with Palestine among the countries showing substantial prevalence. Similar trends were observed in Saudi Arabia, Jordan, and Lebanon [19]. However, many of these studies did not account for the impact of shift patterns or specific workplace stressors.

Shift work is widely recognized as a significant contributor to nurse burnout. Dall'Ora et al. [1] found that 12‐h shifts are associated with higher EE compared to 8‐h shifts. This aligns with the JD–R model, which suggests that burnout results from an imbalance between JDs (e.g., long hours) and available resources (e.g., autonomy, support) [5]. When nurses choose to work longer shifts voluntarily, this autonomy can mitigate burnout—a finding supported by Stone et al. [6], who reported less EE in nurses who had control over their shift lengths.

Beyond shift length, rotating shifts—which involve inconsistent day/night schedules—may pose additional health risks by disrupting circadian rhythms and limiting recovery time, and increased psychological strain has been observed in nurses working night and rotating shifts [8]. Wisetborisut et al. [20] found that nurses working rotating shifts had a 25% burnout rate, compared to 15% among those with fixed schedules. However, rotating shifts remain underexplored in nursing burnout research, particularly in Middle Eastern or conflict‐affected regions.

In the Palestinian context, the evidence is sparse and sometimes contradictory. Hamdan and Hamra [9] reported higher burnout in emergency departments in the West Bank compared to Gaza. In contrast, Abukhader et al. [10] found that fixed day‐shift nurses experienced more EE than those on rotating shifts. These inconsistencies highlight the need to examine how local factors—such as staffing levels, managerial support, and institutional culture—mediate the burnout‐shift work relationship.

Demographic factors also influence burnout. For example, studies by Abukhader et al. [10] and Im et al. [11] noted that younger, unmarried nurses and those without children often report higher burnout, possibly due to limited professional experience or fewer coping mechanisms. Gender differences have also been reported. Rezaei et al. [13] and Cañadas‐De la Fuente et al. [12] found that female nurses often face higher EE due to balancing work with family responsibilities. However, Yektatalab et al. [14] observed that male nurses may exhibit higher levels of DP, suggesting a gendered response to workplace stress.

Although these studies provide valuable insights, most are cross‐sectional and rely on self‐reported data, limiting their ability to draw causal conclusions [15]. Few have explored the combined effect of demographic and organizational factors in politically unstable environments like Palestine, where nurses face not only professional but also sociopolitical stressors.

In light of these gaps, this study aims to investigate the association between rotating shift work and burnout among nurses in Hebron hospitals, while considering socio‐demographic factors such as gender, marital status, and years of experience. This research contributes to a more nuanced understanding of burnout in healthcare settings marked by both systemic and contextual challenges.

### 2.1 Research Question

Does working rotating shifts significantly influence burnout levels among nurses in Hebron hospitals?

### 2.2 Hypothesis

Nurses working rotating shifts experience higher levels of burnout—specifically EE, DP, and reduced PA—compared to those with non‐rotating schedules.

## 3 Methodology

### 3.1 Study Design

This study employed a descriptive cross‐sectional design using a quantitative approach. The aim was to assess the association between rotating shift work and burnout among nurses working in Hebron hospitals, West Bank, Palestine. A cross‐sectional design was chosen to provide a snapshot of burnout levels and related factors at a single point in time.

### 3.2 Study Setting

The study was conducted in four hospitals in Hebron Governorate:

- Two governmental hospitals: Hebron Governmental Hospital and Al‐Muhtaseb Governmental Hospital.
- Two non‐governmental hospitals: Al‐Mezan Hospital and the Palestinian Red Crescent Hospital.

These institutions include a variety of departments, such as intensive care units (ICU), cardiac care units (CCU), emergency rooms (ER), and medical‐surgical wards, allowing for broad coverage of nursing environments.

### 3.3 Study Period

Data collection was carried out between January 2025 and March 2025.

### 3.4 Population and Sampling

The target population included all registered nurses working in the selected hospitals (estimated total = 669 nurses). Due to the unavailability of a complete and up‐to‐date list of nurses, non‐probability convenience sampling was used. The required sample size was calculated on the basis of a 95% confidence level, 5% margin of error, and an estimated 15% prevalence of burnout, yielding a minimum required sample of 152 participants. To account for non‐response and incomplete data, 195 nurses were invited to participate.

Of these, 175 completed the questionnaire (response rate: 89.7%). After excluding incomplete responses, a total of 166 valid questionnaires were included in the final analysis.

### 3.5 Inclusion Criteria

- Registered nurses currently employed in one of the selected hospitals.
- Nurses with at least 1 year of professional experience.

### 3.6 Exclusion Criteria

- Nurses who were on leave during the data collection period.
- Nurses who declined to participate or submitted incomplete responses.

### 3.7 Data Collection Tool

Data were collected using a structured, self‐administered questionnaire consisting of three sections:

1. Socio‐demographic characteristics (e.g., age, gender, marital status, number of children, educational level).
2. Occupational characteristics (e.g., years of experience, hospital type, department, work schedule, overtime, income).
3. Burnout assessment, measured using the Maslach Burnout Inventory–Human Services Survey (MBI‐HSS).

The Arabic version of the MBI‐HSS was used, having been previously translated and reviewed by clinical experts to ensure linguistic and contextual accuracy. The MBI‐HSS includes 22 items across three subscales:

- Emotional Exhaustion (EE),
- Depersonalization (DP), and
- Personal Accomplishment (PA).

Each item was rated on a 5‐point Likert scale (0 = strongly disagree to 4 = strongly agree). The internal consistency of the scale was confirmed using Cronbach's alpha:

- EE: *α* = 0.887,
- DP: *α* = 0.883,
- PA: *α* = 0.930,
- Overall scale reliability: *α* = 0.9301.

### 3.8 Data Collection Procedure

Following ethical approval from the Palestine Polytechnic University IRB (RCK, 220‐2024) and authorization from hospital administrators and the Palestinian Ministry of Health, the questionnaires were distributed manually to nurses during work hours. Participants were informed about the purpose of the study and assured of the confidentiality and anonymity of their responses. Questionnaires were returned in sealed envelopes to ensure privacy.

### 3.9 Ethical Considerations

- Ethical approval was granted by the College of Nursing at Palestine Polytechnic University.
- Written informed consent was obtained from all participants.
- Participation was voluntary, and respondents were allowed to withdraw at any time without any consequences.
- No personal identifiers were collected, and all data were stored securely and used solely for research purposes.

### 3.10 Data Analysis

Data were analyzed using IBM SPSS Statistics version 26. Descriptive statistics (frequencies, percentages, means, and standard deviations) were used to summarize the data. Inferential statistics, including independent *t*‐tests, one‐way ANOVA, and Pearson's chi‐square tests, were used to examine associations between burnout levels and socio‐demographic or occupational variables. A *p* value of less than 0.05 was considered statistically significant.

## 4 Results

From Table 1, the majority of participating nurses were male (58.4%) and under 30 years old (48.2%). Most worked rotating shifts (68%) and were married (70.5%). Notably, 59% had 7 years or less of experience, and 45.2% reported working overtime. Half of the participants were employed in governmental hospitals, with registered nurses comprising the largest job category.

**TABLE 1:** Socio‐demographic and occupational characteristics of participating nurses (*N* = 166).

Variable	Category	Frequency (*n*)	Percentage
Gender	Female	69	41.6
	Male	97	58.4
Age group (years)	20–30	80	48.2
	31–40	60	36.1
	>41	26	15.7
	Mean (SD)		31.2 (6.89)
Work schedule	Day/Morning shift	53	32.0
	Rotating shift	113	68.0
Marital status	Married	117	70.5
	Unmarried	49	29.5
Number of children	None	66	39.8
	1–3 children or more	100	60.2
Financial support to others	No	71	42.8
	Yes	95	57.2
Hospital type	Governmental hospitals	83	50.0
	Non‐governmental hospitals	83	50.0
Current workplace	Government sector	83	50.0
	Non‐government sector	83	50.0
Job title	Head Nurse	18	10.8
	Assistant Head Nurse	15	9.0
	Registered Nurse	93	56.0
	Licensed Practical Nurse (LPN)	34	20.5
	Others (midwifery)	6	3.6
Experience (years)	≤7	98	59.0
	>7	68	41.0
Overtime work	No	91	54.8
	Yes	75	45.2

Table 2 shows emotional exhaustion (EE), depersonalization (DP), and low‐personal accomplishment (lowPA) subscales. For EE, 25.3% of nurses scored low, 50.6% scored moderate, and 24.1% scored high, with a mean score of 22.7 (SD = 7.57). For DP, 50.0% of nurses scored low, 18.7% scored moderate, and 31.3% scored high, with a mean score of 7.02 (SD = 5.59). In the Low‐PA subscale, 95.8% of nurses scored low, 3.0% scored moderate, and 1.2% scored high, with a mean score of 12.5 (SD = 8.29). The overall mean burnout score across all subscales was 42.1 (SD = 17.54), indicating significant burnout levels among the nurses in this study.

**TABLE 2:** Prevalence of burnout among participated nurses (*n* = 166).

		Frequency	Percent	Mean (SD)
Emotional exhaustion (EE)	Low (0–18)	42	25.3	
	Moderate (19–27)	84	50.6	22.7 (7.57)
	High (>27)	40	24.1	
	Total	166	100.0	
Depersonalization (DP)	Low (0–5)	83	50.0	
	Moderate (6–9)	31	18.7	7.02 (5.59)
	High (≥10)	52	31.3	
	Total	166	100.0	
Low‐personal accomplishment	Low (0–27)	159	95.8	
	Moderate (28–31)	5	3.0	12.5 (8.29)
	High (>31)	2	1.2	
	Total	166	100.0	
Total mean (SD)			42.1 (17.54)	

Table 3 shows demographic characteristics and the mean levels of burnout scores and burnout subscales by gender and shift work. Nurses with rotation shift work schedules reported higher levels of burnout compared with non‐shift workers (*p* value 0.187). Female nurses reported higher levels of EE, Low‐PA, and overall burnout than males (*p* value 0.02, 0.037, and 0.012, respectively). Unmarried nurses and those without children experienced a higher DP, Low‐PA, and total burnout compared to their counterparts (*p* value for marital status: 0.028, 0.009, and 0.048; for children: 0.049, 0.019, and 0.048, respectively).

**TABLE 3:** Relationship between burnout three subscales and socio‐demographic characteristics of participants (*n* = 166).

	EE		DP		Low‐PA		Whole scale	
Variables	Mean (SD)	*p* value	Mean (SD)	*p* value	Mean (SD)	*p* value	Mean (SD)	*p* value
Gender								
Female	24.4 (7.3)	0.020	7.9 (5.8)	0.112	13.9 (8.7)	0.037	46.2 (18.6)	0.012
Male	21.6 (7.6)		6.4 (5.4)		11.2 (7.8)		39.3 (16.3)	
Age group								
20–30	22.8 (7.7)	0.992	7.6 (5.7)	0.166	13.3 (8.7)	0.165	43.7 (17.9)	0.271
31 and over	22.8 (7.5)		6.4 (5.5)		11.5 (7.8)		40.7 (17.2)	
Marital status								
Married	22.7 (7.4)	0.914	6.4 (5.5)	0.028	11.3 (7.7)	0.009	40.4 (16.9)	0.048
Unmarried	22.8 (7.9)		8.4 (5.9)		14.9 (9.1)		46.3 (18.4)	
Number of children								
None	23.2 (7.1)	0.566	8.1 (5.9)	0.049	14.2 (8.7)	0.019	45.5 (18.4)	0.048
1–3 children	22.5 (7.9)		6.3 (5.3)		11.1 (7.8)		39.9 (16.7)	
Support others								
No	22.7 (8.3)	0.903	7.4 (5.9)	0.505	12.4 (8.2)	0.928	42.5 (18.8)	0.837
Yes	22.8 (7.1)		6.8 (5.3)		12.3 (8.4)		41.9 (16.7)	
Hospitals								
Hebron Alia Gov Hospital	24.2 (6.4)	0.005	5.5 (4.6)	0.012	10.9 (6.0)	0.007	22.7 (7.6)	0.004
Al‐Muhtaseb Gov Hospital	24.2 (5.3)		7.3 (6.5)		12.6 (9.6)		7.02 (5.6)	
Al‐Mizan Private Hospital	23.6 (8.9)		9.3 (5.4)		16.1 (8.5)		12.4 (8.3)	
Red Crescent Society Hosp	19.4 (8.4)		6.0 (4.9)		10.1 (7.3)		42.1 (17.5)	
Current workplace								
Government	24.2 (5.7)	0.014	6.5 (5.8)	0.257	11.9 (8.3)	0.461	42.6 (16.2)	0.735
NGO and/or private	21.3 (8.8)		7.5 (5.4)		12.8 (8.3)		41.7 (18.9)	
Job Title								
Head Nurse	21.5 (8.3)	0.714	5.2 (3.9)	0.139	10.8 (4.8)	0.324	37.5 (13.5)	0.425
Assistant Head Nurse	22.5 (10.1)		9.5 (4.8)		14.3 (8.7)		46.4 (19.9)	
Registered Nurse	23.3 (7.2)		6.6 (5.9)		11.5 (8.5)		41.5 (17.7)	
Licensed Practical Nurse	22.8 (7.3)		8.1 (5.4)		14.5 (8.5)		45.4 (17.1)	
Others (midwifery)	19.3 (6.5)		6.0 (6.5)		12.2 (10.6)		37.5 (22.1)	
Experience in years								
≤7	23.1 (7.4)	0.539	8.1 (6.5)	0.021	13.5 (8.6)	0.037	44.4 (17.9)	0.047
>7	22.3 (7.8)		10.9 (4.9)		10.8 (7.6)		41.7 (16.7)	
Work schedule/shift								
Others	22.1 (8.3)	0.476	6.1 (5.2)	0.147	11.3 (7.4)	0.220	39.5 (16.9)	0.187
Rotation	23.1 (7.2)		7.4 (5.7)		12.9 (8.7)		43.4 (17.9)	
Overtime work								
No	22.1 (6.8)	0.482	6.8 (5.6)	0.537	12.3 (8.4)	0.980	42.3 (17.5)	0.920
Yes	22.3 (8.4)		7.3 (5.6)		12.4 (8.2)		41.9 (17.7)	

Nurses at Hebron Alia Governmental Hospital had the highest EE and overall burnout scores (*p* values 0.005 and 0.004, respectively), whereas those at the Red Crescent Society Hospital had the lowest. Governmental hospital nurses reported higher EE than those in NGOs or private hospitals (*p* value 0.014). Nurses with less than 7 years of experience showed higher DP, Low‐PA, and overall burnout (*p* values 0.021, 0.037, and 0.047, respectively).

## 5 Discussion

This study explored the prevalence and predictors of burnout among nurses working in Hebron hospitals, with a particular focus on rotating shift schedules. The findings revealed moderate‐to‐high levels of burnout, with significant variations across demographic and organizational factors.

The overall mean burnout score was 42.1 (SD = 17.54), indicating considerable levels of burnout consistent with previous studies in similar settings [9, 20]. EE emerged as the most prevalent dimension, with 50.6% of participants reporting moderate levels and 24.1% reporting high levels. This aligns with reports from El‐Menyar et al. [4] and Giorgi et al. [17], who found that shift work, especially rotating schedules, is associated with elevated EE among nurses.

Female nurses in the present study exhibited significantly higher levels of EE, low PA (Low‐PA), and overall burnout compared to their male counterparts. These findings support previous research suggesting that female nurses may experience higher burnout due to the combined pressures of professional and domestic responsibilities [12, 13]. However, the current results contrast with those of Yektatalab et al. [14], who indicated that male nurses might experience higher DP, highlighting potential gendered variations in the burnout experience that warrant further investigation.

Unmarried nurses and those without children also reported significantly higher DP, Low‐PA, and total burnout scores. These findings are consistent with Abukhader et al. [10] and Im et al. [11], who emphasized that younger, unmarried nurses may lack coping resources or social support systems, increasing their vulnerability to burnout. Similarly, less experienced nurses (≤7 years) demonstrated higher DP, Low‐PA, and overall burnout, reinforcing the role of professional experience as a buffer against occupational stress, as noted by Alidosti et al. [21] and Hamdan and Hamra [9].

The type of healthcare institution was another significant factor. Nurses employed in governmental hospitals, particularly Hebron Alia Governmental Hospital, reported higher EE and overall burnout compared to those in non‐governmental settings. This aligns with previous studies suggesting that resource constraints, higher patient loads, and organizational challenges in public sector facilities may exacerbate nurse burnout [9, 15].

Rotating shift schedules were associated with higher burnout levels, though the difference was not statistically significant. However, nurses working rotating shifts reported higher DP, echoing prior research linking shift rotations to circadian rhythm disruption, fatigue, and psychological strain [8, 20]. These findings underscore the need for further research examining the nuanced impacts of shift rotation on different burnout dimensions.

Contradictory evidence exists within the literature. For example, Abukhader et al. [10] reported higher EE levels among fixed‐day shift nurses compared to those on rotation, whereas Shah et al. [22] highlighted regional and institutional variability in burnout prevalence across healthcare settings. Such inconsistencies may stem from differing healthcare infrastructures, staffing levels, and sociopolitical factors unique to specific regions, including Palestine.

It is important to interpret these results within the framework of the JD–R model [5], which posits that burnout results from an imbalance between JDs (e.g., shift work, workload) and available resources (e.g., autonomy, social support). The current findings suggest that insufficient resources, particularly in governmental hospitals and among less experienced nurses, may amplify the negative impact of JDs, leading to heightened burnout.

Nevertheless, the results must be considered in light of several limitations. The cross‐sectional design precludes causal inferences regarding the relationship between shift work and burnout, as highlighted by Diehl et al. [15]. Additionally, the use of self‐reported measures may introduce response bias, and the convenience sampling approach limits generalizability. Furthermore, the study did not stratify burnout levels by detailed nursing roles, which may have provided deeper insights into occupational stress variations [23].

Despite these limitations, this study contributes valuable data on burnout among Palestinian nurses and highlights the intersection of shift work, demographic characteristics, and organizational factors in influencing burnout. Future longitudinal and mixed‐method studies are recommended to explore causal relationships and incorporate qualitative insights into nurses’ lived experiences of burnout, particularly in politically complex regions like Palestine.

## 6 Conclusion

This study highlights the considerable prevalence of burnout among nurses in Hebron hospitals, with rotating shift schedules, gender, marital status, years of experience, and type of employing institution identified as significant contributing factors. Female nurses, unmarried individuals, those without children, less experienced nurses, and those working in governmental hospitals, particularly Hebron Alia Governmental Hospital, reported higher burnout levels across multiple dimensions.

These findings underscore the need for targeted interventions, particularly in high‐risk groups and settings, to mitigate burnout and promote nurse well‐being. Strategies should focus on optimizing shift schedules, enhancing organizational support, and developing stress management programs. Addressing these factors is essential not only for safeguarding nurses’ mental health but also for ensuring the quality and safety of patient care.

Given the cross‐sectional design and reliance on self‐reported data, future research should adopt longitudinal approaches to better understand causal relationships between shift work and burnout and to evaluate the long‐term effectiveness of intervention strategies.

## 7 Relevance to Clinical Practice

### 7.1 Impact on Nurse Well‐Being

The study highlights the critical issue of burnout among nurses, particularly those working rotating shifts. Recognizing and addressing burnout in clinical practice is essential for maintaining the physical and mental well‐being of nurses, which directly influences their ability to deliver high‐quality patient care.

### 7.2 Workplace Improvements

The findings emphasize the need for healthcare institutions to reassess staffing levels, shift scheduling, and support systems. Implementing measures to reduce the impact of rotating shifts, such as optimizing shift rotation and offering mental health resources, can enhance nurses' job satisfaction and reduce burnout, ultimately improving patient care outcomes.

### 7.3 Policy Implications

The study provides valuable insights for healthcare administrators and policymakers to develop targeted interventions to support nurses, especially those at high risk of burnout. These strategies can improve retention rates, prevent turnover, and ensure a healthier work environment for healthcare professionals in Hebron hospitals and similar settings.

## Author Contributions

Yousef Jaradat contributed to the study design, literature review, data collection, and initial manuscript drafting. Mohammed Qtait (Corresponding Author) supervised the study, led methodology and data analysis, integrated all sections, and finalized the manuscript. Nesreen Alqaissi contributed to methodology refinement, ethical coordination, and manuscript revision. Dana Abo Omer assisted with data collection, transcription, and drafting of the results. Asal Atawneh contributed to literature search, data entry, and preparation of tables and figures. Areen Abu Sharkh supported participant recruitment, data organization, and preliminary coding. Mohammed Baradia contributed to data verification, methodological drafting, and manuscript review. Hamza A. Drabee assisted with validation, coding checks, and proofreading of the final manuscript

## Funding

The authors have nothing to report.

## Conflicts of Interest

The authors declare no conflicts of interest.

## Data Availability

The data that support the findings of this study are available from the corresponding author upon reasonable request.

## References

[1] C. Dall'Ora , O. Z. Ejebu , J. Ball , and P. Griffiths , “Shift Work Characteristics and Burnout Among Nurses: Cross‐Sectional Survey,” Occupational Medicine 73, no. 4 (2023): 199–204, 10.1093/occmed/kqad046. 37130349 PMC10195190

[2] W. B. Schaufeli , “Burnout: A Short Socio‐Cultural History,” in Burnout, Fatigue, Exhaustion, ed. S. Neckel , A. Schaffner , and G. Wagner (Palgrave Macmillan, Cham, 2017).

[3] C. Dall'Ora , J. Ball , A. Recio‐Saucedo , and P. Griffiths , “Characteristics of Shift Work and Their Impact on Employee Performance and Wellbeing: A Literature Review,” International Journal of Nursing Studies 57 (2021): 12–27, 10.1016/j.ijnurstu.2016.01.007. 27045561

[4] A. El‐Menyar , W. H. Ibrahim , W. El Ansari , et al., “Characteristics and Predictors of Burnout Among Healthcare Professionals: A Cross‐Sectional Study in Two Tertiary Hospitals,” Postgraduate Medical Journal 97, no. 1151 (2021): 583–589, 10.1136/postgradmedj-2020-137547. 32796117

[5] M. H. Kim , A. C. Mazenga , X. Yu , et al., “Factors Associated With Burnout Amongst Healthcare Workers Providing HIV Care in Malawi,” PLoS ONE 14, no. 9 (2019): e0222638, 10.1371/journal.pone.0222638. 31550281 PMC6759146

[6] P. W. Stone , C. Mooney‐Kane , E. L. Larson , et al., “Nurse Working Conditions and Patient Safety Outcomes,” Medical Care 45, no. 6 (2007): 571–578, 10.1097/MLR.0b013e3180383667. 17515785

[7] N. V. Shifrin and J. S. Michel , “Flexible Work Arrangements and Well‐Being: A Review and Research Agenda,” Journal of Management 48, no. 3 (2022): 728–758, 10.1177/01492063211064442.

[8] P. Peng , S. Chen , Y. Hao , Y. Zhang , and X. Li , “Network of Burnout, Depression, Anxiety, and Dropout Intention in Medical Undergraduates,” International Journal of Social Psychiatry 69, no. 6 (2023): 1520–1531, 10.1177/00207640231166629. 37092762

[9] M. Hamdan and A. A. A. Hamra , “Burnout Among Workers in Emergency Departments in Palestinian Hospitals: Prevalence and Associated Factors,” BMC Health Services Research 17, no. 1 (2017): 407. 28619081 10.1186/s12913-017-2356-3 PMC5472878

[10] I. Abukhader , K. Abukhader , O. Naser , Y. Saeed , and A. Maliashe , “Burnout Among Palestinian Nurses Working in Governmental and Private Hospitals at Nablus District,” Open Journal of Social Sciences 8, no. 7 (2020): 1–11, 10.4236/jss.2020.87001.

[11] C. Im , S. Song , and K. Kim , “The Associations of Psychological Burnout and Time Factors on Medication Errors in Rotating Shift Nurses in Korea: A Cross‐Sectional Descriptive Study,” Nursing Open 10 (2023): 5550–5559, 10.1002/nop2.1794. 37115503 PMC10333822

[12] G. A. Cañadas‐De la Fuente , E. Ortega , L. Ramirez‐Baena , E. I. De la Fuente‐Solana , C. Vargas , and J. L. Gómez‐Urquiza , “Gender, Marital Status, and Children as Risk Factors for Burnout in Nurses: A Meta‐Analytic Study,” International Journal of Environmental Research and Public Health 15, no. 10 (2018): 2102, 10.3390/ijerph15102102. 30257449 PMC6209972

[13] O. Rezaei , K. Habibi , D. Arab Ghahestany , et al., “Factors Related to Job Burnout Among Nurses in the Razi Psychiatric Hospital, Iran,” International Journal of Adolescent Medicine and Health 32, no. 3 (2018): 20170146, 10.1515/ijamh-2017-0146. 29500920

[14] S. Yektatalab , K. Honarmandnejad , and R. Janghorban , “Relationship Between Occupational Burnout and Demographic Variables Among Nurses in Jahrom, Iran,” Pan African Medical Journal 34, no. 1 (2019): 22, 10.11604/pamj.2019.34.22.15642. 31762891 PMC6859009

[15] E. Diehl , S. Rieger , S. Letzel , et al., “The Relationship Between Workload and Burnout Among Nurses: The Buffering Role of Personal, Social and Organisational Resources,” PLoS ONE 16, no. 1 (2021): e0245798, 10.1371/journal.pone.0245798. 33481918 PMC7822247

[16] H. J. Freudenberger , “Staff Burnout,” Journal of Social Issues 30, no. 1 (1974): 159–165, 10.1111/j.1540-4560.1974.tb00706.x.

[17] F. Giorgi , A. Mattei , I. Notarnicola , C. Petrucci , and L. Lancia , “Can Sleep Quality and Burnout Affect the Job Performance of Shift‐Work Nurses? A Hospital Cross‐Sectional Study,” Journal of Advanced Nursing 74, no. 3 (2018): 698–708, 10.1111/jan.13484. 29164664

[18] Z. Chemali , F. L. Ezzeddine , B. Gelaye , et al., “Burnout Among Healthcare Providers in the Complex Environment of the Middle East: A Systematic Review,” BMC Public Health [Electronic Resource] 19, no. 1 (2019): 1–21, 10.1186/s12889-019-7713-1. 31640650 PMC6805482

[19] I. Elbarazi , T. Loney , S. Yousef , and A. Elias , “Prevalence of and Factors Associated With Burnout Among Health Care Professionals in Arab Countries: A Systematic Review,” BMC Health Services Research 17, no. 1 (2017): 1–10, 10.1186/s12913-017-2319-8. 28716142 PMC5513024

[20] A. Wisetborisut , C. Angkurawaranon , W. Jiraporncharoen , R. Uaphanthasath , and P. Wiwatanadate , “Shift Work and Burnout Among Healthcare Workers,” Occupational Medicine 64, no. 4 (2014): 279–286, 10.1093/occmed/kqu009. 24550196

[21] M. Alidosti , M. Delaram , L. Dehgani , and M. Maleki Moghadam , “Relationship Between Self‐Efficacy and Burnout Among Nurses in Behbahan City, Iran,” Women's Health Bulletin 3, no. 4 (2016): 1–5, 10.17795/whb-30445.

[22] M. K. Shah , N. Gandrakota , J. P. Cimiotti , N. Ghose , M. Moore , and M. K. Ali , “Prevalence of and Factors Associated With Nurse Burnout in the US,” JAMA Network Open 4, no. 2 (2021): e2036469, 10.1001/jamanetworkopen.2020.36469. 33538823 PMC7862989

[23] Y. Jaradat and M. Qtait , “Occupational Stress and Associated Risk Factors Among Nurses in Hebron Hospitals: A Cross‐Sectional Study From the West Bank, Palestine,” SAGE Open Nursing 11 (2025): 23779608251374155, 10.1177/23779608251374155. 40893697 PMC12397598

